# Diet quality and consumption of ultra-processed foods according to age groups in Brazil: insights from the National Dietary Survey 2017–2018

**DOI:** 10.1017/S000711452510411X

**Published:** 2025-09-14

**Authors:** Ariane Cristina Thoaldo Romeiro, Flávia dos Santos Barbosa Brito, Débora Martins dos Santos, Emanuela Santos da Costa, Cintia Chaves Curioni, Amanda Rodrigues Amorim Adegboye

**Affiliations:** 1 Department of Social Nutrition, Institute of Nutrition, Rio de Janeiro State University, Rio de Janeiro, Brazil; 2 Centre for Agroecology, Water and Resilience, Coventry University, Coventry, UK; 3 Centre for Healthcare and Communities, Coventry University, Coventry, UK

**Keywords:** Diet quality, Ultra-processed foods, Food consumption, Sociodemographic factors

## Abstract

Previous studies demonstrated that ultra-processed foods (UPF) affect overall diet quality. However, none have yet examined this relation across different age groups in Brazil. This study assessed the relationship between diet quality and the consumption of UPF in a Brazilian population according to age groups. This was a cross-sectional study that analysed food consumption data from 46 164 Brazilians aged ≥10 years who participated in the 2017–2018 National Dietary Survey. Food and beverages consumed were recorded by two 24-h recalls. All food items were classified as UPF or non-UPF according to the Nova system. Diet quality was evaluated using nutritional density and the prevalence of inadequate nutrient consumption, according to the quintiles of energy contribution of UPF. The association between diet quality and UPF consumption was evaluated by linear and Poisson regressions, with adjustment for sociodemographic variables, stratified by age groups (adolescents, adults and older adults). The consumption of UPF increased the densities of carbohydrates, free sugar, saturated fat and Na and decreased the densities of proteins, fibres and potassium in three age groups. Higher prevalence ratios (PR) of inadequate consumption of free sugar and fibre among the lower and higher quintiles of energy contribution of UPF among adolescents (PR = 2·02, 95 % CI = 1·82, 2·25; PR = 1·88, 95 % CI = 1·68, 2·10), adults (PR = 1·86, 95 % CI = 1·75, 1·98; PR = 1·70, 95 % CI = 1·60, 1·80) and older adults (PR = 1·48, 95 % CI = 1·30, 1·69; PR = 1·24, 95 % CI = 1·09, 1·40). UPF consumption was negatively associated with diet quality across different age groups. Thus, interventions targeting UPF consumption should be implemented across life stages to improve overall diet quality.

Diet quality refers to the assessment of an individual’s overall dietary intake, measured against the foods and nutrients recommended by dietary guidelines, such as national food guides^(1,2)^. A healthy diet is characterised by the consumption of fresh and minimally processed foods, including whole grains, fruits and vegetables. These foods are nutrient-dense, providing essential protective components such as fibre, vitamins and minerals, while containing lower levels of potentially harmful elements including saturated fats, sugars and salt^(3–5)^.

Global shifts in food systems have not only contributed to the weakening adherence to healthy dietary patterns in line with dietary recommendations but also led to an increase in the consumption of ultra-processed foods (UPF)^(6–8)^. According to the Nova classification system, UPF are industrially formulated products that are ready-to-eat and manufactured from the combination of substances extracted or derived from foods along with sensory additives to enhance the taste and palatability of the products^(9)^.

A systematic review demonstrated that diets predominantly composed of UPF have low nutritional quality. This is mainly due to the high densities of harmful nutrients such as saturated fat, sugar and Na and energy found in such foods^(10)^. Therefore, the consumption of UPF has also been related to many negative health outcomes, such as weight gain, as well as the occurrence of several non-communicable diseases^(11–14)^. Considering these aspects, studies evaluating the consumption of UPF are useful for monitoring these dietary risk factors, supporting food and nutrition educational strategies and promoting public policies that encourage healthy eating^(15)^.

International agencies such as the FAO of the UN, the Pan American Health Organization and the International Network for Food and Obesity/Non-communicable Diseases Research, Monitoring and Action Support have suggested the use of energy contribution of UPF (expressed as % of total energy) as an indicator to monitor the nutritional quality of diets^(16–18)^. Studies conducted in Australia, Korea and Belgium examining the relationship between the energy contribution of UPF and the nutritional quality of diets have shown age-related differences, with findings indicating higher consumption of UPF and a decline in diet quality particularly among children and adolescents^(19–21)^.

In Brazil, Louzada *et al.* demonstrated that UPF consumption has a detrimental effect on overall diet quality^(22)^. The 2017–2018 Household Budget Survey (HBS) further revealed that younger individuals have a higher energy intake from UPF^(23)^. However, no studies have yet examined diet quality specifically in relation to UPF consumption across different age groups in Brazil. While population-based research such as the ISA-Capital survey highlighted that adolescents tend to have the poorest diet quality compared with adults and older adults^(24)^, there is still a gap in understanding how UPF consumption affects dietary quality across different age groups. The present study addresses this gap by conducting a stratified analysis by age group, investigating the relationship between UPF consumption and diet quality in adolescents, adults and older adults using data representative of the Brazilian population.

## Methods

### Background

The 2017–2018 HBS is the sixth conducted by the Brazilian Institute of Geography and Statistics (Instituto Brasileiro de Geografia e Estatística – IBGE, in Portuguese), which assesses the structures of consumption, expenditures, income and part of the asset variation of the households, providing a profile of the life conditions of the population based on the analysis of the household budgets^(25)^. IBGE is the official Brazilian Population Statistics Agency and the main provider of data and information about the country. The last two HBS included the Brazilian National Dietary Survey (NDS), which assessed individual food consumption in a subsample of the households surveyed in the HBS, with data from all individuals aged 10 years and over living in the households. The assessment, compilation and structuring of this study counted on the contribution of technicians from the Ministry of Health, which added the vision of Nutrition specialists, of acknowledged experience and skill, who contributed both to the design of the project and the production of the publication, as well as to the data validation up to the final step of the analysis^(23)^.

All these surveys used a secondary database that is publicly and freely available. The microdata, which includes data, documentation, questionnaires, table translators, reading software and calculation memory, is freely available on the IBGE website: https://www.ibge.gov.br/en/statistics/experimental-investigations/experimental-statistics/25610-pof-2017–2018-pof-en.html?edicao=28652&t=microdados. Microdata ensures confidentiality by omitting identifiable information, such as household addresses, telephone numbers and census tract numbers. Brazilian census data are protected by law (Law No. 13,709/2018 – General Data Protection Law). Supplementary Law No. 105/2001), which ensures that confidential information is not made available to the public. The IBGE was conducted in the 2017–2018 HBS and NDS following the ethical principles of the Declaration of Helsinki.

### Study design and sampling

**Figure 1.**
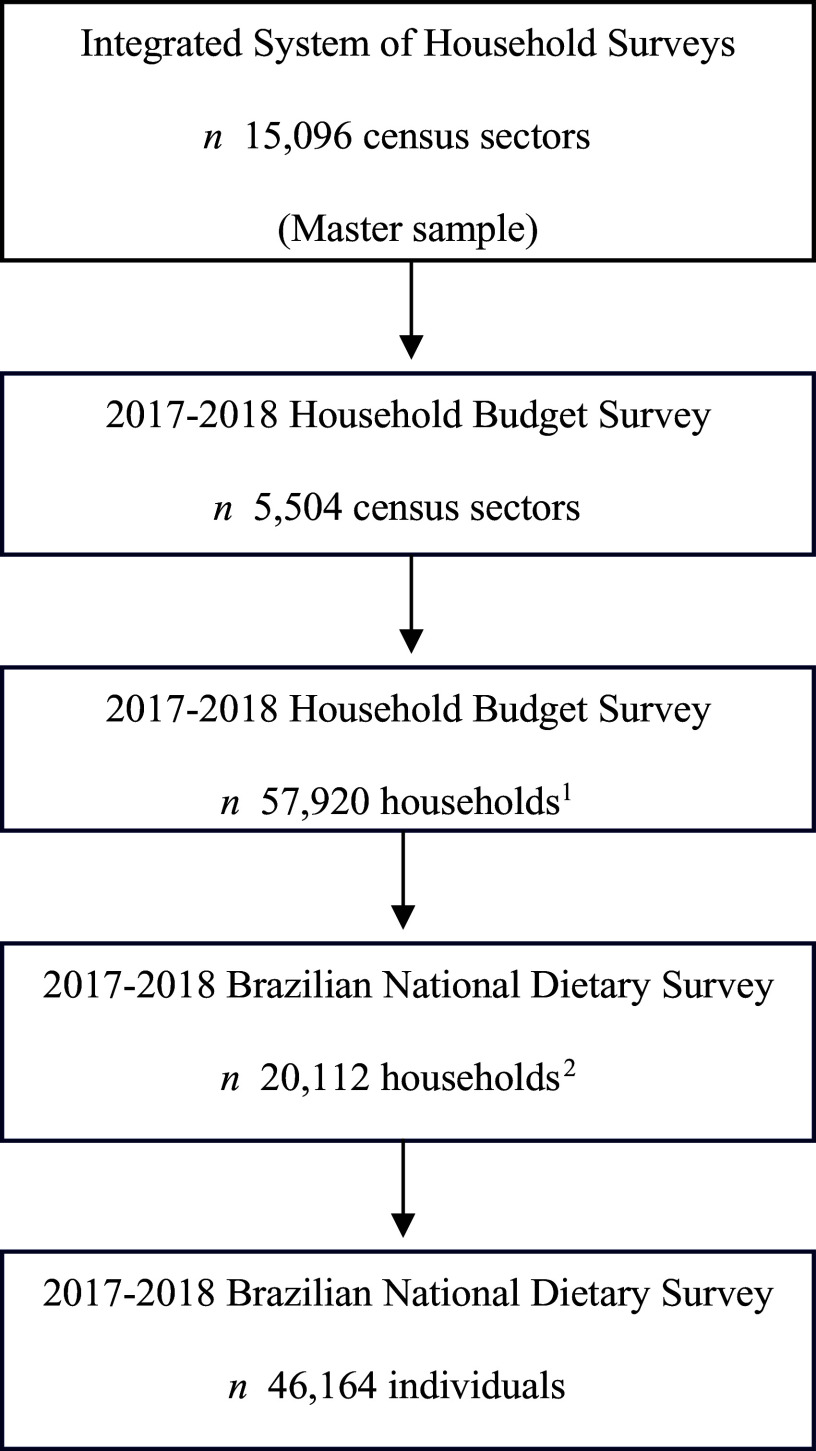
Sample flow chart in 2017–2018 Household Budget Survey and Brazilian National Dietary Survey. ^1^Households randomly selected from the predefined stratification system. An average loss of 15 % was estimated due to possible refusals to answer the survey, and the same proportion was added to the final number of households to minimise possible losses. ^2^Households randomly selected from the predefined stratification system.

This is a cross-sectional study using data from the Brazilian NDS and the HBS, both conducted by the IBGE in 2017–2018. The NDS was conducted simultaneously in a random subsample of 34·7 % of the HBS. The HBS used the IBGE Integrated Household Survey System, which selects a ‘Master Sample’, defined as a set of census tracts that cover the entire national territory. The complex sample was selected in two stages. The Primary Sampling Units (PSU) were derived from the master sample, which consists of a set of census sectors. The PSU were selected by sampling with probability proportional to the number of households in each sector. The census sectors were previously obtained by government administrative divisions, urban or rural settings and income levels based on the Demographic Census of the year 2010. In the second stage, the households were selected by simple random sampling. The sectors were distributed over the four-quarters of the year in which the research was carried out. For the assessment of individual food consumption, a subsample of households was selected from the sample by simple random sampling. The 2017–2018 HBS consisted of 5504 selected sectors and 57 920 investigated households, with 20 112 households randomly selected of the 57 920 households surveyed in the HBS. All individuals aged 10 years and over in the households surveyed in the 2017–2018 HBS were invited to participate in the NDS^(25)^. The final sample included 46 164 individuals aged 10 years or older (Fig. 1). Additional information on the sampling process and the definition of the master sample can be obtained from official publications of the IBGE^(23,25)^.

### Data collection

Data collection was carried out over 12 months, from July 2017 to July 2018. The information collected in the 2017–2018 HBS was based on seven modules. For this study, only the modules 1 and 7 were used: the former assessed sociodemographic characteristics and the latter assessed individual food consumption. Further detailed information about data collection can be found in a previous publication by IBGE^(23,25)^.

### Sociodemographic characteristics

The sociodemographic characteristics considered in this study were sex (male and female), age groups (10–19 years; 20–59 years and 60 years and over), years of education (0–4 years, 5–8 years, 9–11 years and 12 years and above), household per capita income (in quartiles), household arrangement (single-person, couple, couple with children, single parent with children or mixed) and macro-regions (North, Northeast, Southeast, South and Midwest). All these characteristics were answered by the reference person in the household.

Age groups were based on the 2017–2018 NDS and the WHO classification^(23,26)^. Per capita income was estimated by dividing the total family income (estimated from the sum of monthly monetary and non-monetary income of all family members) by the number of individuals in the family. The conversion of the Brazilian currency to US dollars was conducted based on the exchange rate as of 31 January 2018^(27)^. Per capita income was then divided into quartiles including a per capita monthly family income of less than US$ 226 (1st quarter), from US$ 226 to less than US$ 391 (2nd quarter), from US$ 391 to less than US$ 678 (3rd quarter) and equal to or greater than US$ 678 (4th quarter).

The variable ‘household arrangement’ was constructed based on the residents’ relationship, that is, their degree of kinship or nature of the existing subordination with the reference person (who was responsible for the household). The spouse was the resident who lived conjointly with the reference person. The child/children were those considered as legitimate, adopted or brought up by the reference person and/or spouse. The arrangement ‘single parent with children’ was composed of the reference person in the household of both sexes and with at least one child. Mixed households were composed of other members with or without any degree of kinship with the reference person or spouse (e.g. son-in-law, daughter-in-law, parents, grandparents, grandchildren, siblings, domestic workers and other relatives). Regarding the macro-regions of the country, the twenty-six states were divided into five regions as proposed by IBGE (South, South East, North, Northeast and Midwest)^(25)^.

### Individual food consumption

Individual food consumption data were collected using two 24-h recalls on non-consecutive days during the week when a trained IBGE agent was present in the household. This collection process was uniform across the entire sample to ensure representativeness in each quarter of the year and to account for seasonal variations in food consumption.

Both 24-h periods were collected through face-to-face interviews, with an 84 % response rate for both periods. These 24 h were collected using the USDA Automated Multiple-Pass Method (AMPM), which is a structured, computer-assisted, multiple-pass method developed by the US Department of Agriculture (USDA). The AMPM has five steps. Each step guides the respondent through the recall process, prompting them to provide more detailed information and reducing the likelihood of omissions^(28)^.

While the individual reported the data uninterruptedly, the IBGE agent made a list on paper. Another household member could help if one person cannot complete the 24 h. The IBGE developed software to register the time, occasion and place, and to enter all food and beverages consumed in the previous day, including recipes, ingredients, food preparation methods, portion sizes in household measures and items added to foods^(23)^. The software issued an alert if the individual reported fewer than five foods or had not reported any foods for more than 3 h. In these cases, the IBGE agent had to confirm that all foods consumed during the day had been recorded and investigate any omissions or incomplete data. In the final stage, the information was checked for missing data and recording errors. All records with an energy intake of less than 300 kcal or more than 10 000 kcal were checked for consistency^(23)^.

The choice of 24 h was based on the fact that it is almost universally used in population-based research and is considered the method with the lowest possibility of systematic error. Another reason was the results of the instrument validation study conducted in the 2008–2009 NDS, which showed better performance of 24 h than food records using double-labelled water as the gold standard method. Furthermore, IBGE pretested and validated the collection instruments, performed quality control procedures during data collection and deleted inconsistent records and replaced them with imputed values in order to minimise the biases inherent in the use of dietary surveys^(23,29)^.

### Nutritional composition of foods

The food and beverage intake databases contained 1832 items^(23)^. To convert these items into amounts consumed in grams or millilitres, the IBGE used the Table of Reference Measures for Food Consumed in Brazil, developed in the 2008–2009 NDS-HBS, reviewed and updated in the 2017–2018 NDS-HBS^(30)^. The energy and nutrient content of each food item reported in 24 h was obtained from the Brazilian Food Composition Table, version 7.0^(31)^, available at http://www.fcf.usp.br/tbca, in accordance with standards and guidelines for the generation, compilation and use of food composition data of FAO/INFOODS (Food and Agriculture Organization/International Network of Food Data Systems), at https://www.fao.org/infoods/infoods/tables-and-databases/faoinfoods-databases/en/).

### Classification of ultra-processed foods

The Nova food classification system, developed by a team at the University of São Paulo, in Brazil, classifies all foods and food products into four groups according to the nature, extent and purpose of the industrial processing they undergo. It considers all physical, biological and chemical methods used during the food manufacturing process, including the use of additives^(9,15)^. Definitions and lists of examples for each of the four Nova groups can be found at https://www.fsp.usp.br/nupens/en/food-classification-nova/ and in the supplementary material (online Supplementary Table 1).

**Table 1.** Sociodemographic characteristics of the study population: National Dietary Survey, Brazil, 2017–2018 (*n* 46 164)

Characteristics	*n*	%
Sex		
Male	21 460	47·9
Female	24 704	52·1
Age (years)		
10–19	8475	17·8
20–59	29 353	64·6
≥ 60	8336	17·6
Education (years)		
0–4	11 723	20·7
5–8	11 946	23·9
9–11	6015	13·7
≥ 12	16 480	41·7
Per capita income (in US$)		
< 226	11 930	21·9
≥ 226 to < 391	11 594	22·9
≥ 391 to < 678	11 389	25·5
≥ 678	11 251	29·7
Household arrangement		
Single person	2841	6·2
Couple	6117	13·1
Couple with children	20 007	44·9
Single parent with children	4326	9·4
Mixed	12 873	26·4
Region		
North	6836	8·2
Northeast	16 097	27·0
Southeast	11 471	42·7
South	6020	14·5
Midwest	5740	7·7

As the focus of this study was on UPF, all foods and beverages reported were classified as ultra-processed or non-ultra-processed according to the NOVA food classification^(9,15)^. All UPF were further divided into subgroups (sweet biscuits, cakes, and pies; packaged salty snacks; bread; confectionaries; soft drinks and refreshments; milk-based drinks; pizzas, hamburgers, sandwiches, and savouries; frozen or instant ready-made dishes; reconstituted meat products; and others).

All food groupings were based not only on the Nova classification system, but also on the food composition, the list of ingredients, nutritional information, resolutions on norms and standards, food processing technology, and published studies^(22,32,33)^. This process was carried out by two independent researchers. When discrepancies in classification occurred, they discussed these differences until reaching a consensus. In these cases, they resolved the issues by adopting the most conservative classification, that is, the lowest degree of processing. However, there were some exceptions to this approach, including items such as bread, ready-to-eat cereals and salty snacks.

### Diet quality assessment

The energy densities of proteins, carbohydrates, free sugar, total and saturated fats (expressed as % of energy), fibre (g/1000 kcal), potassium (mg/1000 kcal) and Na (mg/1000 kcal) were calculated. Based on nutritional recommendations for healthy diets proposed by the WHO, the inadequate consumption of free sugars and saturated fats (≥ 10 % of total energy), total fats (≥ 30 % of total energy), fibres (≤ 12·5 g/1000 kcal), potassium (< 1755 mg/1000 kcal) and Na (≥ 1 g/1000 kcal) was also assessed^(4,34,35)^. Adjustments were made using the Multiple Source Method (MSM) developed by the European Prospective Investigation Cancer and Nutrition (EPIC)^(36)^. This programme estimates the usual intake of food and nutrients, eliminating intrapersonal variance of consumption^(37,38)^. The MSM was used to estimate usual nutrient intake, free sugar, dietary energy and usual UPF energy intake.

### Data analysis

Sociodemographic characteristics of the study population were described using absolute (*n*) and relative (%) frequencies. The relationship between diet quality and UPF consumption by age group, including adolescents, adults and older adults, was assessed in three ways. First, mean and standard error (se), relative contribution (%) to the total energy and quintiles of energy contribution of UPF and their subgroups were described. Second, average dietary indicators were evaluated according to quintiles (%) of the total energy from UPF foods. Linear regressions were used to examine if there was a trend in average dietary indicators according to quintiles (%) of total energy from UPF, adjusted for sociodemographic characteristics, identified in the literature as possible confounders^(39)^. This approach has been used in most studies examining the effect of UPF on diet quality^(10)^. Third, the prevalence of inadequate nutrient consumption was calculated according to quintiles of energy contribution of UPF. These associations were investigated using a multiple Poisson regression, with robust variance, adjusted for sociodemographic characteristics, identified in the literature as possible confounders^(39)^, providing the prevalence ratio (PR) and corresponding 95 % CI. The Poisson regression was used due to the high prevalence of UFP consumption among the Brazilian population. In this scenario, the PR produces more robust and narrower CI estimates^(40)^. The assumptions of the adjusted regression models were tested using the omnibus test (*P* value < 0·05) and *P*-value for deviance (*P* value > 0·05) to determine the significance and goodness of fit of the models.

All analyses were performed using Stata/SE statistical package version 16 (Stata Corp.), considering the expansion factors, the complexity of the sample design and a significance level of 5 %.

## Results

Out of the 46 164 participants, 52·1 % were women, 64·6 % were adults, 41·7 % of the reference person attended school for at least 12 years, 29·7 % were located in the highest per capita income, 44·9 % were living in a household arrangement composed of a couple with children and 42·7 % were located in the southeast region of Brazil (Table 1).

### Ultra-processed food consumption

The results revealed a significant variation in the energy contribution of UPF foods across age groups. Adolescents exhibited the highest consumption, with their intake ranging from 6·4 % (lowest quintile) to 59·2 % (highest quintile) of their total daily energy intake. Adults followed a similar pattern, albeit with a slightly lower range of 4·6 % to 50·5 %. Older adults demonstrated the lowest consumption, with a range of 3·7 % to 42·9 % of their energy intake derived from UPF. All age groups displayed an upward trend in UPF consumption across the quintiles (Table 2).

**Table 2.** Average of absolute and relative consumption and contribution (%) of the total energy of ultra-processed foods and their subgroups according to age groups: National Dietary Survey, Brazil, 2017–2018 (*n* 46 164)

	UPF	Sweet biscuits, cakes and pies	Packaged salty snacks	Bread^a^	Confectionaries^b^	Soft drinks and refreshments	Milk-based drinks	Pizzas, hamburgers, sandwiches and savouries	Frozen or instant ready-made dishes^c^	Reconstituted meat products^d^	Others^e^
Adolescents^†^											
Mean kcal	563·0	344·7	236·7	205·4	232·5	150·2	231·3	352·0	454·7	161·2	112·7
se	8·5	9·0	6·7	9·2	9·6	3·0	4·7	8·3	15·5	8·2	1·9
% of total energy	27·4	4·7	2·7	1·0	2·0	2·5	2·4	5·9	1·5	1·4	3·4
1st Q	6·4	0·6	0·9	0·2	0·2	0·5	0·2	0·4	0·1	0·4	2·9
2nd Q	13·6	1·6	1·9	0·5	0·5	1·4	0·9	1·4	0·1	0·7	4·5
3rd Q	22·5	2·9	2·5	1·3	1·1	2·6	2·5	3·2	0·6	1·4	4·4
4th Q	34·8	6·1	3·5	1·6	2·6	3·3	3·8	6·6	1·7	2·1	3·6
5th Q	59·2**	11·6**	5·0**	1·6**	5·1**	4·7**	4·5**	16·6**	4·5**	2·3**	3·2*
Adults^‡^											
Mean kcal	433·8	251·0	184·4	174·7	219·3	143·5	196·9	359·8	462·0	167·1	103·2
se	5·1	5·3	3·7	3·5	5·5	2·0	3·9	7·5	13·6	4·7	1·4
% of total energy	20·3	1·9	1·9	1·3	1·4	1·8	0·7	5·4	1·3	1·1	3·4
1st Q	4·6	0·3	0·5	0·2	0·2	0·4	0·1	0·2	0·1	0·3	2·4
2nd Q	9·8	0·7	1·3	0·7	0·3	1·0	0·2	0·9	0·1	0·5	4·2
3rd Q	16·0	1·3	1·8	1·3	0·8	1·8	0·6	2·4	0·2	1·0	4·6
4th Q	25·9	2·5	2·7	2·3	1·8	2·5	1·2	5·8	1·1	1·7	4·3
5th Q	50·5**	5·3**	3·8**	2·4**	1·8**	4·1**	1·8**	17·5**	5·0**	2·4**	4·2**
Older adults^§^											
Mean kcal	310·8	172·8	143·8	146·6	204·0	113·5	173·7	332·2	393·4	134·0	76·2
se	7·0	6·8	4·7	4·3	11·9	4·5	7·7	23·9	20·8	6·6	1·8
% of total energy	16·4	1·5	1·9	1·8	1·2	1·0	0·6	3·4	1·1	0·7	3·2
1st Q	3·7	0·3	0·6	0·3	0·1	0·2	0·0	0·1	0·0	0·2	1·8
2nd Q	8·4	0·7	1·2	0·8	0·3	0·6	0·3	0·4	0·0	0·3	3·8
3rd Q	13·4	1·2	2·2	1·7	0·7	0·9	0·4	1·2	0·1	0·6	4·3
4th Q	21·2	1·9	2·9	3·0	1·4	1·4	0·8	3·9	0·8	1·0	4·2
5th Q	42·9**	4·0**	3·7**	4·0**	4·0**	2·2**	1·5**	12·3**	4·8**	1·9**	4·5**

Q, Quintiles of energy proportion from total UPF intake in the total energy intake; UPF, ultra-processed foods.

a Bread and toast.

b Candies, lollipops, desserts, ice cream and chocolates.

c Pasta dishes, instant noodles, powdered mixtures for soups and mash.

d Sausages, nuggets, poultry and fish finger.

e Soya products, distilled alcoholic beverages, ready-made sauces, margarine, cheeses and artificial sweeteners.

*P value < 0·05.

**P value < 0·001 for linear trend adjusted for sex, age (years), income, education (years), household arrangement and macro-region.

† 1st Q: 0–9·7; 2nd Q: 9·8–17·3; 3rd Q: 17·4–27·7; 4th Q: 27·8–42·8; 5th Q: 42·9–100.

‡ 1st Q: 0–7·2; 2nd Q: 7·3–12·3; 3rd Q: 12·4–19·9; 4th Q: 20·0–32·9; 5th Q: 33·0–100.

§ 1st Q: 0–6·2; 2nd Q: 6·3–10·5; 3rd Q: 10·6–16·5; 4th Q: 16·6–26·7; 5th Q: 26·8–100.

### Dietary quality and ultra-processed food

The analysis of the nutritional quality of the diets of adolescents, adults and older adults showed a significant decreasing tendency in the average levels of protein, fibre and potassium intake and an increasing tendency in the energy densities of carbohydrates, free sugars, total fats (except for the older adults), saturated fat and Na as the energy contribution of UPF increased. It is noteworthy that the diets of the three age groups had high Na content and low potassium content in all energy contribution strata of UPF (Table 3).

**Table 3.** Average diet indicators according to quintiles (%) of the total energy of ultra-processed foods by age groups: National Dietary Survey, Brazil, 2017–2018 (*n* 46 164)

	Energy (kcal/day)	Protein (% of total energy)^a^	Carbohydrate (% of total energy)^b^	Free sugar (% of total energy)^c^	Total fat (% of total energy)^d^	Saturated fat (% of total energy)^e^	Dietary fibre (g/1000 kcal)^f^	Potassium (mg/1000 kcal)^g^	Na (mg/1000 kcal)^h^
Adolescents^†^									
Overall	1820·2	17·5	53·1	14·4	29·4	9·3	12·4	1175·0	1454·4
1st Q	1672·2	20·1	51·5	10·1	28·4	8·6	15·4	1314·9	1370·8
2nd Q	1792·2	18·3	52·6	13·0	29·0	8·9	13·1	1205·3	1421·7
3rd Q	1873·2	17·3	53·2	15·0	29·5	9·4	12·1	1167·7	1454·1
4th Q	1885·5	16·3	53·8	16·7	29·8	9·5	11·5	1144·0	1485·6
5th Q	1945·4	14·4*	54·8*	19·1*	30·8*	10·4*	9·0*	988·6*	1571·2*
Adults^‡^									
Overall	1786·2	18·6	51·6	11·7	29·8	9·4	13·0	1291·9	1463·7
1st Q	1712·1	20·7	50·3	8·9	29·0	8·9	15·0	1403·3	1362·2
2nd Q	1786·3	19·1	51·4	10·5	29·5	8·9	13·9	1321·4	1418·6
3rd Q	1801·5	18·3	51·9	12·1	29·8	9·1	12·8	1284·5	1449·4
4th Q	1803·4	17·7	52·5	13·3	29·8	9·5	12·1	1271·0	1496·1
5th Q	1878·7	15·9*	52·9*	15·5*	31·2*	10·6*	9·9*	1111·2*	1648·6*
Older adults^§^									
Overall	1543·2	18·4	52·8	10·1	28·8	9·3	13·8	1406·4	1428·6
1st Q	1487·5	19·8	52·2	8·7	28·0	8·9	15·1	1479·6	1331·9
2nd Q	1588·3	18·9	52·4	8·8	28·7	9·0	14·4	1416·7	1406·4
3rd Q	1572·7	18·0	52·8	10·0	29·2	9·1	13·7	1388·3	1424·8
4th Q	1536·1	17·7	53·3	11·3	29·0	9·4	13·0	1412·9	1476·5
5th Q	1584·4	16·3*	54·0*	12·6*	29·7	10·3*	11·5*	1278·9*	1575·2*

Q, Quintiles of energy proportion from total UPF intake in the total energy intake; UPF, ultra-processed foods.

a–h , WHO recommended values for proteins (10–15 % of total energy), carbohydrates (55–75 % of total energy), free sugar (< 10 % of total energy), total fats (15–30 % of total energy), saturated fat (< 10 % of total energy), fibre (> 12·5 g/1000 kcal), potassium (> 1·755 mg/1000 kcal) and Na (< 1 g/1000 kcal).

*P value < 0·001 for linear trend adjusted for sex, age (years), income, education (years), household arrangement and macro-region.

† 1st Q: 0–9·7; 2nd Q: 9·8–17·3; 3rd Q: 17·4–27·7; 4th Q: 27·8–42·8; 5th Q: 42·9–100.

‡ 1st Q: 0–7·2; 2nd Q: 7·3–12·3; 3rd Q: 12·4–19·9; 4th Q: 20·0–32·9; 5th Q: 33·0–100.

§ 1st Q: 0–6·2; 2nd Q: 6·3–10·5; 3rd Q: 10·6–16·5; 4th Q: 16·6–26·7; 5th Q: 26·8–100.

When comparing adolescents in the last quintile of energy contribution of UPF with those in the first quintile, higher PR of inadequate free sugar consumption (PR_adj_ = 2·02, 95 % CI = 1·82, 2·25), total fats (PR_adj_ = 1·21, 95 % CI = 1·08, 1·35) and fibre (PR_adj_ = 1·88, 95 % CI = 1·68, 2·10) were observed. Adults in the last quintile of energy contribution of UPF compared with those in the first quintile had inadequate intake of free sugar, increased by 86 % (PR_adj_ = 1·86, 95 % CI = 1·75, 1·98), of total fats, increased by 10 % (PR_adj_ = 1·10, 95 % CI = 1·03, 1·17), and fibres, increased by 70 % (PR_adj_ = 1·70, 95 % CI = 1·60, 1·80). On the other hand, older adults in the highest quintile of energy contribution of UPF compared with those in the first quintile had inadequate intake of free sugar, increased by 48 % (PR_adj_ = 1·48, 95 % CI = 1·30, 1·69), and fibres, increased by 24 % (PR_adj_ = 1·24, 95 % CI = 1·09, 1·40) (Table 4).

**Table 4.** Inadequate nutrient intake according to quintiles of energy contribution of ultra-processed foods by age groups: National Dietary Survey, Brazil, 2017–2018 (*n* 46 164)

		1st Q	2nd Q		3rd Q		4th Q		5th Q	
Adolecents^*^, ^†^										
Free sugar (≥ 10 % of energy)	Prevalence (%)	39·6	57·3		67·1		79·3		85·9	
	PR_adjusted_ (95 % CI)	1·0	1·43	1·28, 1·61^b^	1·63	1·46, 1·82^b^	1·91	1·72, 2·12^b^	2·02	1·82, 2·25^b^
Total fat (≥ 30 % of energy)	Prevalence (%)	44·1	44·5		48·4		51·6		56·8	
	PR_adjusted_ (95 % CI)	1·0	0·99	0·88, 1·12	1·04	0·92, 1·17	1·11	0·99, 1·25	1·21	1·08, 1·35^b^
Saturated fat (≥ 10 % of energy)	Prevalence (%)	96·5	99·5		99·9		100·0		100·0	
	PR_adjusted_ (95 % CI)	1·0	1·03	1·01, 1·04^b^	1·03	1·02, 1·04^b^	1·03	1·02, 1·04^b^	1·03	1·02, 1·04^b^
Dietary fibre (≤ 12·5 g/1000 kcal)	Prevalence (%)	38·6	47·4		54·7		64·1		77·4	
	PR_adjusted_ (95 % CI)	1·0	1·22	1·08, 1·38^b^	1·38	1·22, 1·57^b^	1·58	1·40, 1·77^b^	1·88	1·68, 2·10^b^
Na (≥ 1 g/1000 kcal)	Prevalence (%)	88·4	95·1		96·3		94·3		94·7	
	PR_adjusted_ (95 % CI)	1·0	1·07	1·03, 1·10^b^	1·07	1·04, 1·11^b^	1·05	1·01, 1·09^a^	1·05	1·01, 1·09^a^
Potassium (≤ 1755 mg/1000 kcal)	Prevalence (%)	94·6	97·6		98·1		99·2		99·5	
	PR_adjusted_ (95 % CI)	1·0	1·03	1·01, 1·05^a^	1·04	1·01, 1·06^b^	1·05	1·03, 1·07^b^	1·06	1·03, 1·08^b^
Adults^*,^^‡^										
Free sugar (≥ 10 % of energy)	Prevalence (%)	37·5	44·6		55·0		61·3		73·3	
	PR_adjusted_ (95 % CI)	1	1·16	1·09, 1·25^b^	1·42	1·33, 1·52^b^	1·57	1·47, 1·67^b^	1·86	1·75, 1·98^b^
Total fat (≥ 30 % of energy)	Prevalence (%)	45·7	46·1		50·2		53·3		56·9	
	PR_adjusted_ (95 % CI)	1	0·97	0·91, 1·03	1·03	0·96, 1·09	1·07	1·00, 1·14^a^	1·10	1·03, 1·17^a^
Saturated fat (≥ 10 % of energy)	Prevalence (%)	97·4	99·4		99·9		99·9		99·7	
	PR_adjusted_ (95 % CI)	1	1·01	1·01, 1·02^b^	1·02	1·01, 1·03^b^	1·02	1·01, 1·03^b^	1·02(	1·01, 1·02^b^
Dietary fibre (≤ 12·5 g/1000 kcal)	Prevalence (%)	38·8	42·5		51·0		57·0		71·5	
	PR_adjusted_ (95 % CI)	1	1·08	1·01, 1·15^a^	1·27	1·19, 1·36^b^	1·4	1·31, 1·49^b^	1·7	1·60, 1·80^b^
Na (≥ 1 g/1000 kcal)	Prevalence (%)	89·2	92·5		95·6		95·7		95·6	
	PR_adjusted_ (95 % CI)	1	1·03	1·01, 1·05^b^	1·06	1·05, 1·08^b^	1·07	1·05, 1·08^b^	1·07	1·05, 1·09^b^
Potassium (≤ 1755 mg/1000 kcal)	Prevalence (%)	90·4	92·8		94·3		95·5		97·3	
	PR_adjusted_ (95 % CI)	1	1·03	1·01, 1·05^b^	1·05	1·03, 1·07^b^	1·07	1·05, 1·08^b^	1·09	1·09, 1·11^b^
Older adults^*,^^§^										
Free sugar (≥ 10 % of energy)	Prevalence (%)	38·8	42·5		45·3		49·4		56·5	
	PR_adjusted_ (95 % CI)	1	1·1	0·96, 1·26	1·19	1·04, 1·36^a^	1·29	1·13, 1·48^b^	1·48	1·30, 1·69^b^
Total fat (≥ 30 % of energy)	Prevalence (%)	42·5	44·5		47·3		50·5		51·3	
	PR_adjusted_ (95 % CI)	1	0·99	0·88, 1·12	1·01	0·89, 1·14	1·06	0·94, 1·19	1·04	0·91, 1·18
Saturated fat (≥ 10 % of energy)	Prevalence (%)	97·8	99·3		99·8		99·9		99·7	
	PR_adjusted_ (95 % CI)	1	1·01	1·00, 1·02^b^	1·01	1·01, 1·02^b^	1·01	1·01, 1·02^b^	1·01	1·00, 1·02^b^
Dietary fibre (≤ 12·5 g/1000 kcal)	Prevalence (%)	42·2	39·8		42·4		48·0		55·3	
	PR_adjusted_ (95 % CI)	1	0·94	0·82, 1·07	0·99	0·87, 1·12	1·09	0·97, 1·24	1·24	1·09, 1·40^b^
Na (≥ 1 g/1000 kcal)	Prevalence (%)	85·9	91·4		94·2		94·0		94·4	
	PR_adjusted_ (95 % CI)	1	1·05	1·02, 1·09^a^	1·08	1·05, 1·12^b^	1·09	1·05, 1·13^b^	1·09	1·06, 1·13^b^
Potassium (≤ 1755 mg/1000 kcal)	Prevalence (%)	87·3	90·0		88·9		92·1		90·8	
	PR_adjusted_ (95 % CI)	1	1·03	1·00, 1·07	1·02	0·99, 1·06	1·07	1·03, 1·10^b^	1·06	1·02, 1·10^a^

Q, quintiles of energy proportion from total UPF intake in the total energy intake; PR adjusted, prevalence ratios adjusted for sex, age (years), income, education (years), household arrangement and macro-region; UPF, ultra-processed foods.

All models were tested for goodness of fit using the omnibus test (*P* value < 0·05) and *P* value for deviance (*P* value > 0·05).

a *P* value < 0·05.

b *P* value < 0·001.

* The usual consumption estimated by the Multiple Source Method.

† 1st Q: 0–11·4; 2nd Q: 11·5–18·3; 3rd Q: 18·4–25·9; 4th Q: 26·0–36·0; 5th Q: 36·1–82·5.

‡ 1st Q: 0–7·6; 2nd Q: 7·7–13·3; 3rd Q: 13·4–19·3; 4th Q: 19·4–27·9; 5th Q: 28·0–81·7.

§ 1st Q: 0–6·6; 2nd Q: 6·7–11·4; 3rd Q: 11·5–16·7; 4th Q: 16·8–24·1; 5th Q: 24·2–80·2.

## Discussion

The present study found that in the 2017–2018 NDS, UPF accounted for approximately 27 % of the total energy intake for Brazilian adolescents, 20 % for adults and 16 % for older adults. As consumption of UPF increased across all age groups, there was a significant decline in the average intake of essential nutrients like protein, fibre and potassium. Conversely, there was a concerning rise in energy density, carbohydrates (particularly free sugars), total fats (except for older adults), saturated fats and Na as the energy contribution of UPF increased.

Adolescence represents a critical developmental period marked by significant behavioural changes, including the adoption of potentially unhealthy habits such as excessive screen time, increased consumption of meals away from home and decreased family meal participation^(41,42)^. These emerging patterns can establish long-term lifestyle trajectories with lasting health implications. Particularly concerning is the high intake of UPF in this population, which may adversely affect adolescent growth and development while potentially establishing detrimental dietary patterns that persist into adulthood^(5)^. A previous study by Crisóstomo *et al.* ^(43)^ investigated UPF consumption within age groups in a Northeastern Brazilian capital city and found that adolescents had the highest intake (26·4 %), while the older adults consumed the least (17·5 %), with results similar to ours. It is worth noting that younger people are the priority target of marketing and advertising strategies for these foods, and therefore, compared with adults and older adults, they are more likely to try most UPF^(44,45)^. Other factors such as convenience, lack of culinary skills and cost may be also important promoters of consumption of these foods among younger individuals^(46)^. Recent evidence also suggests that UPF may induce addictive biological and behavioural responses, consequently driving excessive eating patterns and contributing to obesity, supporting the need for future research^(47)^.

Our results are also consistent with a previous national study utilising HBS data, which identified a significant inverse relationship between UPF consumption and the dietary content of essential nutrients like protein, fibre, vitamins and minerals^(22)^. These findings are particularly relevant as a reduction in the dietary share of UPF holds the potential to significantly improve overall dietary quality.

Our findings indicate that the types of UPF consumed were similar across all three age groups. Pizza, hamburgers, sandwiches, savoury snacks, soya products, distilled alcoholic beverages, ready-made sauces, margarine, cheeses and artificial sweeteners were the primary sources of energy. Additionally, sweet biscuits, cakes and pies were significant contributors for adolescents and adults, whereas packaged salty snacks and bread held prominence among older adults. Several of these items are consistent with dietary patterns observed in other countries. For instance, breads, fast-food snacks and salted biscuits are noted in Canada^(48)^, while breads, biscuits, cakes and industrial desserts are prevalent in Portugal^(49)^. A study conducted in Brazil showed that rice, coffee, beans, salt, bread and beef are the most commonly consumed foods, with noticeable variations among teenagers. This highlights the importance of further investigating age-related differences in diet quality^(50)^.

The stratification of age groups by quintiles of energy contribution of UPF revealed that the average consumption of these foods can be up to 60 %, 50 % and 43 % of the total energy consumed in the last quintile by adolescents, adults and older adults in the last quintile, respectively. These values were substantially lower than those found in studies conducted in high-income countries^(51,52)^. In Australia, for example, the average consumption of these foods accounted for 84 %, 72 % and 65 % of the total energy consumed by adolescents, adults and older adults, respectively^(51)^. The affordability, convenience and aggressive marketing may be responsible for Australian diets based on UPF^(19)^. Whereas in Brazil, fresh and minimally processed foods, especially traditional items of national cuisine, such as rice, beans, roots and tubers, along with dishes derived from these staples still represent the basis of the Brazilian diet^(23,50)^.

Regarding the analysis of diet quality, most nutritional indicators found in the first quintile of energy contribution of UPF of the diet of adolescents, adults and older adults followed the values recommended by the WHO, except for Na (high) and potassium (low). A possible explanation for these results is that Na intake in the country is quite high, mainly due to the high amounts of cooking salt and salt-based condiments used in culinary preparations^(53)^. We emphasise that, although the nutritional profile of diets with lower energy contribution of UPF was healthier, there is no amount considered safe for the intake of these products, as they tend to have an unbalanced nutritional content^(54)^.

Our findings suggest that as the proportion of UPF in the diets of all age groups increases, the nutritional quality tends to decline, with many indicators deviating significantly from WHO recommendations. This trend is consistent with both national and international research^(10)^. A systematic review with meta-analysis of data from representative national surveys revealed a significant increase in free sugar, total fat and saturated fat content, alongside a decrease in proteins, fibres and several micronutrients including potassium, Mg, vitamin C, vitamin D, Zn, phosphorus, vitamin B_12_ and niacin, as UPF consumption increases^(10)^. These findings align with our own and support previous studies.

In contrast to the previous national survey based on NDS 2008–2009 data, our findings revealed a direct correlation between Na consumption and the increasing presence of UPF in the diets of adolescents, adults and older adults^(55)^. This discrepancy may arise from variations in the types of UPF subgroups consumed, as documented in different editions of the NDS. Specifically, our study noted a significant rise in the intake of items associated with higher Na density across quintiles of UPF energy contribution. Similar trends of increasing Na density with greater consumption of UPF were observed in studies from Chile, Canada and the USA^(56–58)^. Another possible factor is the variation in dietary data collection methods between the two surveys. While the HBS 2008–2009 employed a food record, the HBS 2017–2018 utilised the 24 h. This change was predominantly influenced by the widespread adoption of the 24 h method in population-based research due to its perceived lower susceptibility to systematic errors. Nonetheless, prior research demonstrated that harmonisation analysis strategies enable the assessment of food consumption trends across different survey methods^(29)^.

This study also demonstrated that all age groups with high consumption of UPF, when compared with their counterparts, presented statistically significant increased prevalence in the excessive consumption of free sugar, total fats, saturated fat and Na and insufficient dietary fibre and potassium. Much evidence, including systematic reviews, shows that excessive consumption of free sugar contributes to diets with higher energy density and increases the risk of obesity, type 2 diabetes and dental caries^(59,60)^, while high consumption of fats and Na and low consumption of fibre are positively associated with unfavourable outcomes associated with cardiometabolic disorders and mortality^(14,61–63)^.

In the case of Brazilian older adults, the consumption of these foods may represent a potential risk to their health conditions, given the high prevalence of chronic diseases among this population^(64)^. In addition, there is further evidence indicating that consumption of UPF can cause damage to the quality of life and health of older people, which is associated with a decrease in telomere length, a biological marker of cell ageing^(65)^, with increased risk of developing frailty syndrome^(66)^ and a higher likelihood of having abdominal obesity^(67)^, dyslipidemia^(68)^ and decline in renal function^(69)^.

Based on the above, several countries have implemented strategies to guide adequate and healthy food choices, such as the development of dietary guidelines, standards for front-of-package labelling of foods, and food and nutrition education activities; structuring of tax policies (taxing of sweetened beverages); and adoption of regulatory advertising measures. Food Guides in several countries have adopted the NOVA classification system to establish their recommendations on healthy eating and emphasise that UPF should be avoided^(5,70–73)^.

Brazil recently introduced new front-of-package nutritional labelling standards for UPF, highlighting high levels of critical nutrients like sugar, Na and unhealthy fats, enabling consumers to easily identify unhealthy products^(74,75)^. Chile pioneered this approach, showing significant reductions in UPF purchases^(76)^. Fiscal policies, such as Mexico’s tax on high-energy foods and sugary drinks and Barbados’ tax on sweetened beverages, have also led to decreased consumption^(77,78)^. In Brazil, voluntary agreements to reduce Na and sugar in foods have faced criticism for lack of transparency, weak goals and no penalties for non-compliance^(79–81)^. Although these measures aim to reduce dietary risks, they may not guarantee healthier products and could inadvertently promote UPF consumption, undermining efforts to encourage minimally processed foods^(5,81)^. Additionally, regulating marketing and advertising, especially among children and adolescents, remains crucial, with challenges emerging in digital media regulation^(82)^.

This study has several limitations and strengths. One primary constraint lies in the underreporting of consumed items, inherent to the 24-h method, potentially impacting dietary and nutritional estimates. However, the inclusion of the AMPM during data collection enhances standardisation and systematisation, mitigating interview bias and enhancing data accuracy. This method was also used during the data analysis stage to estimate usual food and nutrient intake, thereby eliminating the effect of intra-individual variation in day-of-week consumption. Misclassification errors could result in either underestimation or overestimation of UPF consumption. To minimise these errors, research teams cross-reviewed each other’s classifications and resolved discrepancies through discussion until consensus was reached. In cases of uncertainty, a conservative approach was adopted, favouring the lower degree of processing or assuming a homemade recipe, which may have led to an underestimation of UPF intake.

Finally, some of the survey data used in this study were collected several years ago. While these represent the most recent nationally representative data available, dietary patterns may have evolved since 2018. Analyses from a prior study, involving data from 2018 to 2022, found an increase in unprocessed or minimally processed foods and processed culinary ingredients prices (R$ 15·3/kg in 2018 to R$ 22·5/kg in 2025), and a decrease in UPF prices (R$ 22·1/kg in 2018 to R$ 20·2/kg in 2025), which may have encouraged the replacement of traditional meals for the consumption of unhealthy foods^(83)^.

Furthermore, the findings are only applicable to individuals aged 10 years and older and do not encompass younger Brazilian children. Nonetheless, utilising a recent database representing the Brazilian population’s food consumption is advantageous. Additionally, adjustments made to estimate usual food consumption reduce errors in estimating inadequate nutrient intake.

Moreover, this study provides valuable insights into UPF consumption at a national level and, to the best of our knowledge, offers the first assessment of age group differences in such consumption.

## Conclusion

Our study corroborates previous findings indicating that UPF consumption may detrimentally affect diet quality, and we observed differences in the dietary quality of adolescents, adults and the elderly, even descriptively, highlighting the unique dynamics of each age group. Therefore, we advocate for nutrition education initiatives and limited access to UPF to promote diet quality and healthy dietary habits.

## Supporting information

Romeiro et al. supplementary material Romeiro et al. supplementary material

## Supplementary material

For supplementary material/s referred to in this article, please visit https://doi.org/10.1017/S000711452510411X
