# Supplementary material for: Diet quality and consumption of ultra-processed foods according to age groups in Brazil: insights from the National Dietary Survey 2017–2018
Source: Br J Nutr. 2025 Aug 22;134(5):413–24. doi: 10.1017/S000711452510411X (PMC12580969; doi:10.1017/S000711452510411X)
Supplement: Romeiro et al. supplementary material [file S000711452510411Xsup001.docx]

TABLE 1. **Nova food classification system and its four groups according to the extent and purpose of food processing**

| **NOVA GROUP** | **DEFINITION** | **EXAMPLES** |
| --- | --- | --- |
| **Non-ultraprocessed foods** | **Unprocessed or minimally processed foods**  *Unprocessed foods:* edible parts of plants (such as fruits, seeds, leaves, stems, roots, and tubers) or animals (including muscle, organs, eggs, and milk), as well as fungi, algae, and water, once they have been separated from their natural environment.  *Minimally processed foods:* unprocessed foods that have undergone industrial procedures like the removal of inedible or unwanted parts, drying, crushing, grinding, fractioning, roasting, toasting, boiling, pasteurization, refrigeration, freezing, packaging, vacuum sealing, non-alcoholic fermentation, and other techniques. These processes largely preserve the natural structure of the food and do not involve the addition of salt, sugar, oils, fats, or other substances. Their primary purpose is to extend shelf life, facilitate storage, and often simplify or diversify food preparation. Additives are typically unnecessary in minimally processed foods and are only used in rare cases. | Fresh, squeezed, chilled, frozen, or dried fruit and leafy and root vegetables; grains (rice, corn cob or kernel, wheat); legumes (beans, lentils, and chickpeas); starchy roots and tubers (potatoes, and cassava); fungi (fresh or dried mushrooms); meat, poultry, fish and seafood; eggs; milk; fruit or vegetable juices (sugar-free); grits, flakes or flour made from grains or roots; nuts (salt or sugar-free); herbs and spices (thyme, oregano, mint, pepper, cloves and cinnamon); plain yoghurt; tea, coffee, and drinking water; granola (cereals or nuts, sugar-free), honey or oil; pasta, couscous and polenta (flours, flakes or grits and water); foods with vitamins and minerals added (wheat or corn flour fortified with iron and folic acid). |
|  | **Processed culinary ingredients**  These are substances derived directly from unprocessed foods or natural sources through industrial techniques such as pressing, centrifugation, extraction, refining, dehydration, or mining. Additives are generally unnecessary and rarely present in processed culinary ingredients. They are primarily used to prepare, season, and cook meals from unprocessed or minimally processed foods. | Vegetable oils (from seeds, nuts or fruit, with or without added anti-oxidants); butter and lard (from milk and pork, with or without salt); sugar (from cane or beet); honey (from combs and syrup from maple trees); starches (from corn and other plants); salt (from seawater, and with added drying agents or micronutrients, such as iodine). |
|  | **Processed foods**  These are relatively simple food products that are industrially manufactured by combining at least one processed culinary ingredient (like salt, sugar, oil, or fat) with unprocessed or minimally processed foods. Preservation techniques such as canning, bottling, and, in the case of items like bread and cheese, non-alcoholic fermentation and cooking methods like boiling or baking are used. The goal of these processes and ingredients is to extend the shelf life of unprocessed or minimally processed foods and enhance their taste, texture, or appearance. Processed foods may include additives that help preserve the product, maintain its original qualities, or inhibit microbial growth (e.g., preservatives and antioxidants), but they do not contain additives intended solely for cosmetic purposes. | Includes all vegetables and legumes preserved in brine (canned or bottled), salted or sweetened nuts and seeds, fruits stored in syrup, and dried or canned fish.  Also includes breads, cheeses, pastries, cakes, cookies (biscuits), sweet or savory snacks, cured meats, and ready-to-heat items like burgers, pre-prepared pies, pasta, and pizza — provided these are made solely from unprocessed or minimally processed foods combined with salt, oil, sugar, or other processed culinary ingredient, and do not contain additive classes used for cosmetic purposes. |
| **Ultra-processed foods (UPF)** | These are industrially produced food items composed of multiple ingredients (formulations), typically containing high levels of sugar, oils, fats, and salt—often in greater amounts than those found in processed foods. They also include food substances that are rarely or never used in home cooking, such as high-fructose corn syrup, hydrogenated oils, modified starches, and protein isolates. Unprocessed or minimally processed foods are either absent or present only in minimal quantities.  The production of UPF involves advanced industrial methods like extrusion, molding, and pre-frying, along with the addition of various additives. These additives are used not just for preservation but to enhance flavor, appearance, and texture—examples include artificial flavors, colorings, non-sugar sweeteners, and emulsifiers. Packaging is often elaborate and made from synthetic materials.  The aim of these processes and ingredients is to develop highly profitable, convenient, and palatable products—ones that use inexpensive ingredients, have a long shelf life, and are heavily marketed. These foods serve as ready-to-eat or ready-to-drink alternatives to other NOVA food groups and to freshly prepared meals.  UPF can be distinguished from processed foods by the inclusion of ingredients not typically found in home kitchens. These include various forms of sugars (e.g., fructose, high-fructose corn syrup, fruit juice concentrates, invert sugar, maltodextrin, dextrose, lactose), modified oils (e.g., hydrogenated or interesterified oils), protein derivatives (e.g., hydrolyzed proteins, soy protein isolate, gluten, casein, whey protein, mechanically separated meat), and additives with cosmetic purposes (e.g., flavorings, flavor enhancers, colorants, emulsifiers, sweeteners, thickeners, and agents for anti-foaming, bulking, carbonating, gelling, and glazing). | Includes all carbonated soft drinks; reconstituted fruit juices and fruit-flavored beverages; cocoa-based and other flavored dairy drinks, energy drinks, and flavored yogurts; candies and other confectionery products; margarines; processed poultry and fish products like nuggets and sticks; sausages, hot dogs, luncheon meats, and other reconstituted meat items; plant-based meat alternatives; extruded breakfast cereals; powdered instant soups, noodles, and desserts; infant formulas and follow-on milks; as well as "health" and "slimming" items such as meal-replacement shakes and powders.  Also includes breads, pastries, cakes, cookies (biscuits), sweet or savory snacks, cured meats, and ready-to-heat foods like burgers, pre-packaged pies, pasta, and pizza—when these are made using ingredients not commonly found in traditional cooking and/or include additive classes with cosmetic purposes. |
